# Effect of Bizhongxiao decoction and its dismantled formulae on IL-1 and TNF levels in collagen-induced arthritis in rat synovial joints

**DOI:** 10.1186/1742-4682-9-47

**Published:** 2012-11-20

**Authors:** Ya-jing Guo, Jiang Chen, Xin-gui Xiong, Dan Wu, Hao Zhu, Qing-hua Liang

**Affiliations:** 1 Institute of Combined Traditional Chinese and Western Medicine, Xiangya Hospital, Central South University, Changsha, Hunan, 410008, P. R. China; 2 Center of Telemedicine, Xiangya Hospital, Central South University, Changsha, Hunan, 410008, P. R. China

**Keywords:** Bizhongxiao decoction, Rheumatoid arthritis, Interleukin-1, Tumor necrosis factor

## Abstract

**Background:**

Rheumatoid arthritis (RA), a chronic autoimmune disease, affects sufferers in many different ways. Treatment of this chronic condition is particularly challenging. Traditional Chinese Medicine (TCM) provides alternatives. Bizhongxiao decoction (BZX) is a TCM complex, which has been used clinically for many years to treat RA. The purpose of this study is to compare the effects of BZX decoction and its dismantled formulae on IL-1 and TNF-1 levels in rats with RA, and to elucidate its mechanism of action.

**Methods:**

Ninety healthy normal female SD rats were randomly divided into six groups: normal (control), model, BZX decoction, and the three dismantled formulae (I: heat-clearing and detoxication, II: dissipating dampness, and III: blood circulation promotion). Apart from the normal (control) group, the rats in each group were injected subcutaneously with bovine type II collagen and complete Freund adjuvant to establish a collagen-induced arthritis model, so that inhibition of foot swelling in the rats by BZX decoction and its dismantled formulae could be observed. Immunohistochemistry was used to assess the levels of the inflammatory cytokines IL-1 and TNF in synovial joints at various time points.

**Results:**

Twenty-one days after the model was established, the levels of TNF and IL-1 were significantly higher in the model group, BZX decoction group and dismantled formula groups I, II and III than in the normal controls (P < 0.05). The levels of these cytokines were significantly higher in the model group than the BZX decoction or the three dismantled formula groups (P <0.01). At longer times, the TNF and IL-1 levels in model group rose gradually; those in the BZX decoction and dismantled formula groups were gradually reduced. The cytokine levels in the BZX decoction group were lower than in the three dismantled formula groups and continued to decline.

**Conclusions:**

BZX decoction and the three dismantled formulae examined down-regulated the inflammatory factors IL-1 and TNF in collagen-induced arthritis rat models, but BZX exerted the strongest effect.

## Background

Rheumatoid arthritis (RA), a chronic autoimmune disease, affects sufferers in many different ways. RA can start in any joint, but it most commonly begins in the smaller joints of the fingers, hands, and wrists. Other joints that are commonly affected include hips, knees, ankles, feet, neck, shoulders, and elbows. Joint involvement is usually symmetrical, both sides of the body being affected equally. In addition to joint pain and inflammation, people with RA may suffer fatigue, occasional fevers, and a general sense of not feeling well [1,2].

This type of arthritis is usually triggered by a viral infection or by an autoimmune malfunction that can be attributed to many different causes. Treatment of this chronic condition is particularly challenging because it is a systemic disease that usually affects multiple joints, and a variety of immunocompetent cells, cytokines, and inflammatory mediators are activated and participate in the pathogenic process. Interleukin-1 (IL-1) and tumor necrosis factor-alpha (TNF) play key roles in the abnormal immune response [3,4]. As it progresses, RA can result in a number of maladies including bone deformity. The treatment will always include physical therapy, occupational therapy and other strategies to enable the patient to cope with this debilitating disease. Any approach to choosing the correct medication for RA must involve the participation of a medical professional. The seriousness of the condition coupled with the potentially agonizing pain suffered by the patient calls for more potent medicine than can be offered over the counter. Moreover, other types of prescription medication may be required to combat other systems affected by the disease or the viral infection that triggered the condition in the first place. Choosing the appropriate RA medication can be difficult owing to the range of factors that contribute to the development of the disease. Medications used include disease-modifying anti-rheumatic drugs (DMARDs) such as Methotrexate, Sulfasalazine, Leflunomide, Hydroxychloroquine sulfate and Azathioprine. These can also be described as slow-acting anti-rheumatic drugs (SAARDs). They suppress inflammation and may also retard the development of joint erosions. The exact reason why they are effective is not currently well understood.

Non-steroidal anti-inflammatory drugs (NSAIDs) are prescribed as pain killers and include ibuprofen and naproxens. They may also ameliorate inflammatory forms of arthritis such as RA. They have no effect on the progress of the disease but may relieve symptoms. The use of NSAIDs is often limited because they increase the risk of upper gastrointestinal problems such as gastric ulcers. COX-2-specific inhibitors such as celecoxib are also non-steroidal anti-inflammatory agents. They are effective in reducing inflammation and relieving pain and are far gentler on the stomach than the conventional, older NSAIDs. However, coxibs may be associated with an increased risk of cardiovascular events. Corticosteroids such as prednisone and Prednisolone reduce inflammation and suppress the immune system. These agents are used in the treatment of RA, both as tablets and as injections into the affected joint. Prednisolone is sometimes used in moderate to severe RA where NSAIDs and DMARDs fail to control the disease. Biological agents, a new category of arthritis treatments called tumor necrosis factor (TNF) inhibitors, have been developed. Infliximab (otherwise Remicade) is a TNF inhibitor available for the treatment of RA in selected patients. It slows the progression of the disease and reduces joint injury. It is given along with Methotrexate. There is also promising work in the field of stem cell research, indicating that new cartilage and bone tissues can be developed inside the patient to repair damaged joints; however, this work is still at an early stage of development.

Bizhongxiao decoction (BZX) is prescription for RA with which our department has experience. Many years of clinical practice have shown that it is effective against RA in the long term and has no significant side effects [5,6]. In this study, we investigated the mechanism of action of BZX and its dismantled formulae in RA treatment and its effect on the IL-1 and TNF levels in a type II collagen-induced rat arthritis model.

## Materials and methods

### Materials and reagents

Ninety healthy female SD rats aged 45-50 days, body weight 180 ± 20 g, were provided by the Experimental Animal Center of Xiangya Medical School. Complete Freund's adjuvant, bovine type II collagen, and IL-1 and TNF kits were purchased from AB Company (China). Para formaldehyde, PBS, 18 cm stainless steel pressure cooker, bath, electric oven, dip wax cup, and slicer were provided by our laboratory.

BZX consisted of *Hedyotis diffusa, Herba sarcandrae, Semen coicis, Savia miltiorrhiza*, Chinese star jasmine stem, *Rhizoma drynariae*; Heat-clearing and detoxication formula (dismantled group I) consisted of *Hedyotis diffusa, Herba sarcandrae, Radix actinidiae*; Dissipating dampness formula (dismantled group II) consisted of *Semen coicis*, Chinese star jasmine stem, *Viciaamoena* Fisch; Blood circulation promotion formula (dismantled group III) consisted of *Savia miltiorrhiza, Herba sarcandrae* and *Radix actinidiae*.

Chinese medicine dosage for the rats (200 g) was calculated in proportion to humans (70 kg) using body surface area conversion; each gavage was 18.6 g/kg d. All crude drugs were purchased from Central South University Xiangya Hospital pharmacy and the quality agreed with the People's Republic of China Pharmacopoeia (2005). The four Chinese medicine compounds were processed as follows: they were mixed, soaked for 1 h, and boiled twice in a multi-function extraction tank (volume of water added was 8 or 10 times the crude medicine, boiled for 1.5 h or 1 h, respectively). The two filtrates were merged and then evaporated to a thick extract, vacuum dried, crushed to a dry extract powder, dispensed, placed in a vacuum dryer, sealed and stored in a cool place.

### Animal grouping

Using a random number table, the animals were divided into six groups (n = 15 in each): normal (control) group, model group, Bizhongxiao decoction group, dismantled group I, dismantled group II, dismantled group III.

### Experimental animal arthritis model

According to the method of Deng Anmei [7], 90 SD rats, all female, age 40 ~ 50 d, body weight (180 ± 10 g) received food *ad libitum* and were housed with a 12 h/12 h light/dark cycle (exposure time 6:00 ~ 18:00) at 20 ± 3°C for one week to allow them to adapt to the environment. Background noise was 40 ± 10 db. Fifteen rats were randomly selected as the normal (control) group; the remaining 75 were used for modeling. Bovine type II collagen (CII) (10 mg) was mixed with 5 mL of 0.1 mol L^-1^ acetic acid, giving a CII concentration of 2 g L^-1^. The solution was kept at 4°C in a refrigerator overnight. For the experiments, it was mixed 1:1 with Freund's complete adjuvant in an ice bath, ground and emulsified, and 0.25 mL of the resulting suspension (CII concentration = 1 g L^-1^) was injected intradermally into the rat in the left rear foot, the back, and the base of the tail. To enhance the reaction, the injections were repeated at day 21, this time into the right rear foot, the back, and the base of the tail.

### Delivery methods

From day 15 of the experiment, either BZX extract or one of the three dismantled formulae was administered orally to the rats in the four treatment groups, and this treatment continued daily by gavage for 14 days. The other groups were gavage with distilled water. All the rats were fed normally and had free access to water.

### Arthritis index scores

After paw swelling appeared, the arthritis index (AI) scores were assessed every four days by five independent judges [8]. These scores indicate the degree and range of joint swelling and deformation. A five-point scale was used to calculate the AI. 0: no swelling; 1: toe joint swelling; 2: toe joint and toe swelling; 3: paw edema below the ankle; 4: paw swelling including the ankle. The scores for individual limbs and joints were summed to obtain the final total score; the higher the AI score, the more serious the joint symptoms.

### Synovial tissue immunohistochemistry

The rats in each of the six study groups were randomly divided into three sub-groups. On days 14, 21, 28 after the initial CII injection, the rats in one subgroup of each group were killed and the knee and ankle joints were carefully stripped. Synovium sections were fixed with 10% neutral formalin and paraffin-embedded for HE staining and immunohistochemical observation. The sections were examined in accordance with the kit instructions for testing. Three fields (× 400) selected for each tissue slice were used for Bio Mias computer software image processing; the signal strength was analyzed to calculate the total amount of positive staining of the target.

### Statistical analysis

All data were expressed as mean ± SD. A single factor analysis of variance and a Kronbach α test were applied to the different groups. SPSS 13 software was used for statistical analysis.

## Results

### General observation

The CII model rats after the initial injection ate less and the average body weight was reduced by 30 ± 10 g. Later, the fur grew dull and the ankle and toe joints developed various degrees of swelling; the first onset was in the hind legs. All 75 CII-injected rats showed symptoms of arthritis. On day 7 after the initial injection, some joint swelling and ecchymosis was observed and mild swelling occurred in the left hind paw. On day 10 the ankle toe joints and the left hind paw swelling gradually worsen, and some of the articular surface skin appeared shiny and congested. At day 21 after re-injection, the arthritis symptoms of the rats worsened and swelling was observed in the right hind paw. Up to day 28, some of the rats showed limited joint activity and the hind paw swelling increased. One week after treatment began, the joint swelling was gradually reduced in the BZX group; after two weeks, the shiny and congested areas of skin had shrunk slightly, there was decreased reddening of the toes, and the fur was less dull than in the model group.

### Assessment of arthritis index scores

**Figure 1:**
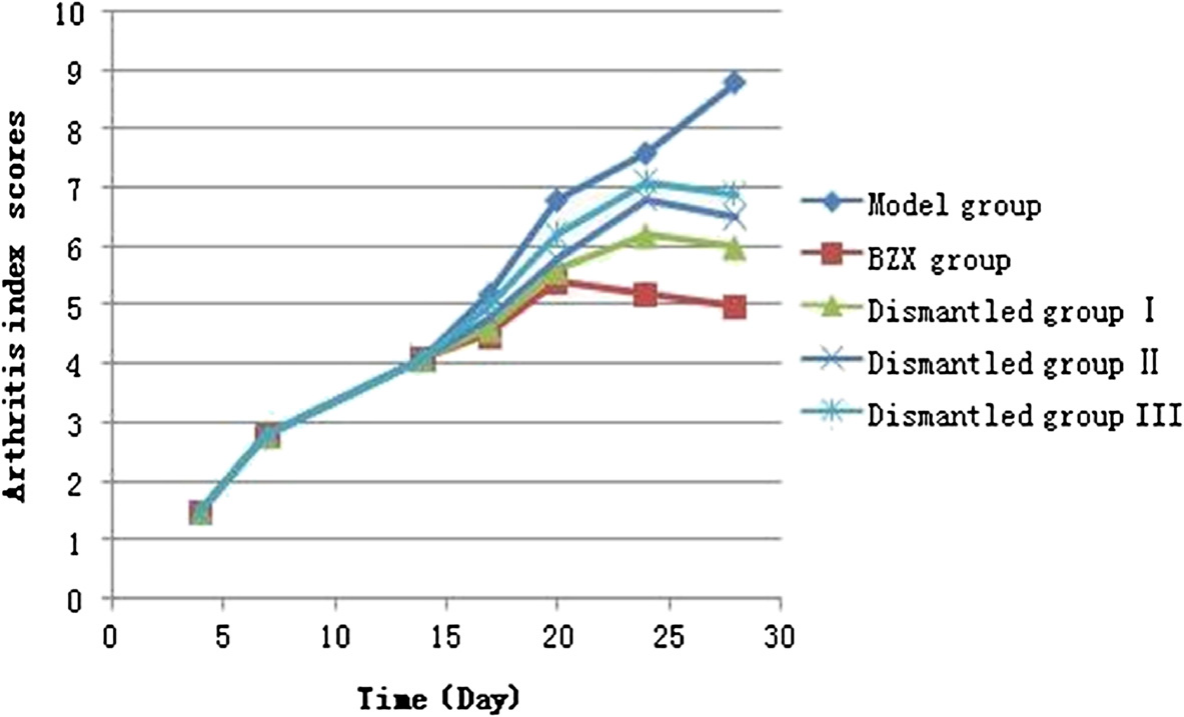
Model group, the BZX Group, dismantled group I, II, III arthritis index scores.

Figure 1 showed the arthritis index scores of model group, the BZX Group, dismantled group I, II, III. There were no significant differences in AI scores among the five CII-injected groups during the first 14 days following the initial injection. The score of the model group continued to increase and reached a peak on day 28 following the initial injection. The scores in the three dismantled groups also reached peaks (20 ± 5 days) but they were lower than the model group peak, and the AI score gradually decreased thereafter. The score for the BZX rats reached its peak at d 20, still lower than the model group, and then showed a gradual steady decline so that it was lower at each time point than the scores for the dismantled groups. On day 28, when the joint inflammation index was lowest, the difference was significant (P < 0.05).

### Synovial tissue TNF expression and effects in the BZX and dismantled groups

**Figure 2:**
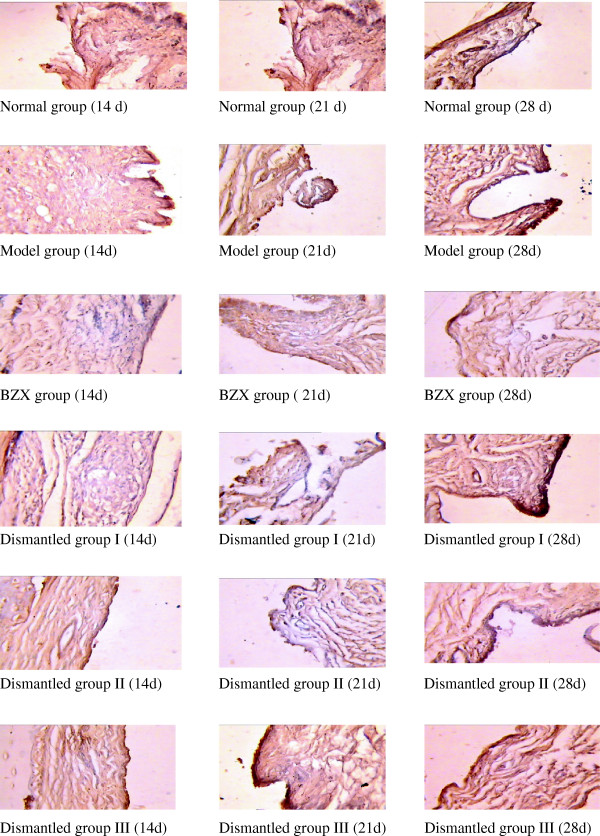
Immunochemical slices after immunication with TNF in AA rats.

Table 1 shows that the serum TNF in the model group followed an increasing trend and was significantly higher than that in the control group (P < 0.05); the rats in the model group showed an obvious inflammatory response. In the BZX and the three dismantled groups, the serum TNF was down-regulated and significantly lower than that in the model group (P < 0.05); the down-regulation was more obvious in the BZX group. TNF expression in the BZX group at days 21, 28 was lower than in dismantled groups I, II and III (P < 0.05) but still higher than in the normal control group (P < 0.05). The TNF level in dismantled group I was lower than that in dismantled group II, which in turn was lower than that in dismantled group III (P < 0.05). These findings suggested that the treatment outcome should be in the order dismantled group I > dismantled group II > dismantled group III. Figure 2 shows the success of the rat RA model and the greater or lesser down-regulation of TNF in the BZX and dismantled groups.

**Table 1:** TNF expression in collagen-induced arthritic rat synovial tissue (mean ± SD)

**Groups**		**TNF**	
	**14d**	**21d**	**28d**
Normal	180 ± 44	180 ± 44	180 ± 44
Model	180 ± 44*	368 ± 81*	485 ± 93*
Bizhongxiao	178 ± 45*	208 ± 28 *	103 ± 24*
Dismantled I	180 ± 43*	248 ± 74 *	197 ± 37*
Dismantled II	177 ± 39*	296 ± 24*	205 ± 45*
Dismantled III	181 ± 41*	312 ± 13*	268 ± 28*

Note: *compared with normal group, P < 0.05; and compared to the same point in time the model group, P < 0.05; compared to prior time points among groups, P < 0.05; compared to the same time points with dismantled formula groups, P < 0.05.

### IL-1 expression in experimental rat synovial tissue, and effects in the BZX and dismantled groups

From Table 2, serum IL-1 in the model group increased with time and was significantly higher than that in the control group (P < 0.05), again indicating an obvious inflammatory response. In the BZX and the three dismantled groups, the serum IL-1 was down-regulated and significantly lower than that in the model group (P < 0.05); the down-regulation was more obvious in the BZX group. IL-1 expression in the BZX group at days 21, 28 was lower than in dismantled groups I, II and III (P < 0.05) but still higher than in the normal control group (P < 0.05). The IL-1 level in dismantled group I was lower than that in dismantled group II, which in turn was lower than that in dismantled group III (P < 0.05). These findings suggested that the treatment outcome should be in the order dismantled group I > dismantled group II > dismantled group III. Figure 2 shows the success of the rat RA model and the greater or lesser down-regulation of IL-1 in the BZX and dismantled groups.

**Table 2:** IL-1 expression in collagen-induced arthritic rat synovial tissue (mean ± SD)

**Groups**		**IL-1**	
	**14d**	**21d**	**28d**
Normal	226 ± 29	226 ± 29	226 ± 29
Model	227 ± 29*	384 ± 73*	483 ± 43*
Bizhongxiao	227 ± 17*	277 ± 79*	248 ± 40*
Dismantled I	226 ± 28*	288 ± 28*	263 ± 20*
Dismantled II	227 ± 29*	315 ± 38*	283 ± 20*
Dismantled III	227 ± 25*	333 ± 36*	308 ± 28*

Note: *compared with normal group, P <0.05; and compared to the same point in time the model group, P <0.05; compared to prior time points among groups, P <0.05; compared to the same time points with dismantled formula groups, P <0.05.

## Discussion

Rheumatoid arthritis (RA) is a systemic autoimmune disease whose cause is not yet fully elucidated, the main symptoms being chronic synovitis and structural joint injury. The current study suggests that cytokines play an important role in the pathogenesis of RA. Cytokines are a class of biologically active substances synthesized and secreted by immune cells and some non-immune cells receiving the appropriate stimulation, and they modulate various cell physiological functions. Studies have confirmed that in RA, large amounts of cytokines are secreted by synovial cells and by monocytes/macrophages and lymphocytes that infiltrate the synovial tissue. These cytokines form a complex network by acting on a variety of cells and adjusting each other’s levels. Imbalance in this network promotes the occurrence and development of RA [9]. Interleukin-1 (IL-1) and TNF play a decisive role in the progression of RA [10]. There are two isoforms of IL-1, IL-1α and IL-1β, IL-1β being instrumental in cartilage damage in RA. Its main role is to promote the proliferation and differentiation of synovial cells and lymphocytes, stimulating synovial cells and cartilage cells to synthesize and release prostaglandin E_2_ (PGE_2_) and collagenase, causing synovial inflammation and disintegration of the cartilage matrix [11,12]. In addition, IL-1 can stimulate synovial cells and cartilage cells to synthesize excess metalloproteinases, but it also promotes the release of Phospholipase A_2_ from synovial cells and chondrocytes, inhibits the synthesis of proteoglycan precursor glycosaminoglycans, and inhibits the mitotis-promoting activity of various growth factors on the chondrocytes, thereby enhancing the destruction of the cartilage matrix. Of the TNF isoforms, TNF-α and TNF-β, TNF-α is most closely implicated in RA. It has a similar role to IL-1, stimulating absorption of the cartilage proteoglycan and inhibiting its synthesis, enhancing the degradation of cartilage, and stimulating synovial cells and cartilage cells to synthesize PGE_2_ and collagenase to cause absorption of bone and cartilage destruction [13]. TNF promotes the formation of osteoclasts, increases their activity, and prolongs their survival, thereby contributing to focal bone erosion in RA. The biological effects of TNF and IL-1 are coordinated and these cytokines act synergistically in most cases. IL-1 is related to the destruction of articular cartilage while TNF-α is mainly involved in the regulation of inflammation [14].

This study demonstrates that on day 25 after modeling, significant joint swelling appeared, and TNF and IL-1 levels were significantly increased and continued to increase gradually with the progress of the disease. After BZX treatment, the TNF and IL-1 levels decreased and the joint swelling showed various degrees of relief, demonstrating the importance of inhibiting these two cytokines in RA treatment.

RA belongs to the "Arthralgia" category in Chinese medicine, but is also called "Arthromyodynia" and "joint-running wind". Its specific etiology and dialectical type pathogenesis are not entirely uniform. Those involved in its management are more focused on the basis of traditional Chinese medicine theory combined with their own clinical experience for differential diagnosis and treatment [15-17].

RA is mostly considered to belong to the same areas of medicine as rheumatic fever, paralysis, the pathogenesis of viscera injuries. Yin and yang imbalance is the inherent basis, and the preconditions are caught cold, hot and humid, toxin disease outside. Toxicity, heat, moisture, and blood stasis are generally believed to be the pathological factors. After a complex pathological process, these factors result in toxin accumulation, heat production, phlegm blockage, blockage of the meridian and final formation of the disease.

**Figure 3:**
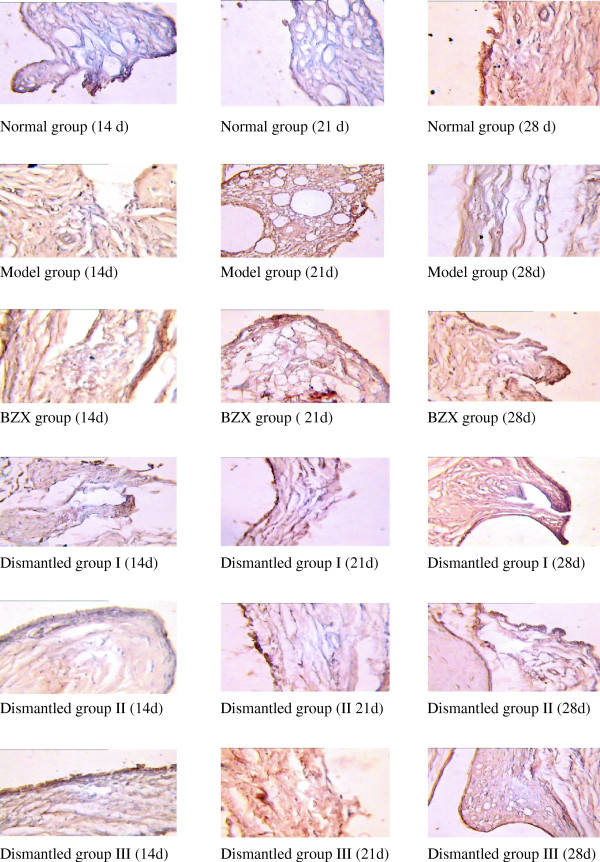
Immunochemical slices after immunication with IL-1 in AA rats.

BZX is produced by Professor Liang Qing-hua for the long-term treatment of active RA. Previous work suggested it significantly inhibits vascular endothelial growth factor expression in affected rat synovial joints [18] and regulates T cell subsets [19]; treatment of active RA with BZX had a good outcome, improving the patients’ morning stiffness, joint pain, joint swelling, and joint tenderness more rapidly than Methotrexate [20]. The rats in the experiment described in the present paper were in the active stage of RA development, and the serum IL-1 and TNF levels were reduced to different degrees in the BXZ group and dismantled groups. The cytokine levels were sufficiently reduced in the BXZ group to approach the normal range; the dismantled groups showed a lesser effect. Synergistic actions among these groups, which affect different aspects of the inflammatory disorder, are obvious. In active RA, heat and toxicity are the main factors. This study indicates that BZX has heat-clearing and detoxication effects, promotes blood circulation, eliminating wind and dredging channels, dissipating dampness for detumescence, and regulating cytokines in order to diminish inflammation in the rats, gradually reducing joint injury. It was then clear that the anti-inflammatory effect of BZX was closely related to the inhibition of inflammatory cytokine production. BZX slows or blocks the proinflammatory effects of inflammatory cytokines and decreases the inflammatory cytokine content of synovial tissue. It decreases the abnormal secretion of IL-1 and TNF, thus reducing inflammation, accelerates the clearance of the inflammatory cytokines, inhibits synovial cell proliferation, and inhibits activated B cells and T cells so as to reduce inflammation and destruction of the synovial tissue. Thus it interrupts the pathological process of RA in rats, and in turn affects the occurrence and development of inflammation. This study further reveals the role of the anti-inflammatory effect of BZX in the experimental rats. Figure 3 showed the results of immunochemical slices after immunication with IL-1 in AA rats. How BZX regulates the network linking the cytokines to each other and to their inhibitors in the experimental rats will be further studied in future experiments.

## Competing interests

The authors declare that they have no competing interest.

## Authors’ contribution

GY-j responsible for experiment. CJ responsible for animal experiment. XX-g responsible for immunohistochemical detections. WD responsible for pathological slices and examination. ZH responsible for pathological slices and examination. LQ-h: Correspondent, responsible for directions and writing. All authors read and approved the final manuscript.
